# Bioderived ionic liquid-based pretreatment enhances methane production from *Agave tequilana* bagasse†

**DOI:** 10.1039/d0ra01849j

**Published:** 2020-04-07

**Authors:** José A. Pérez-Pimienta, José P. A. Icaza-Herrera, Hugo O. Méndez-Acosta, Victor González-Álvarez, Jorge A. Méndoza-Pérez, Jorge Arreola-Vargas

**Affiliations:** Department of Chemical Engineering, Universidad Autónoma de Nayarit Tepic Mexico; Departamento de Ingeniería Química, CUCEI-Universidad de Guadalajara Guadalajara Jalisco Mexico; Department of Engineering in Environmental Systems, Instituto Politécnico Nacional Mexico City Mexico; División de Procesos Industriales, Universidad Tecnológica de Jalisco Guadalajara Jalisco Mexico jorgearreolav85@gmail.com

## Abstract

In recent years, bioderived ionic liquids have gained attention as a new promising approach for lignocellulosic biomass pretreatment. In this work, *Agave tequilana* bagasse (ATB), an attractive bioenergy feedstock in Mexico, was pretreated with a bioderived ionic liquid (cholinium lysinate) for the first time. Optimization of the pretreatment conditions, in-depth biomass characterization and methane generation *via* anaerobic digestion are the main contributions of this work. The results indicated optimized pretreatment conditions of 124 °C, 205 min and 20% solids loading by applying a central composite design. The optimized pretreated ATB was able to produce an elevated sugar yield of 51.4 g total sugars per g ATB due to their high delignification (45.4%) and changes in their chemical linkages although an increase in cellulose crystallinity was found (0.51 untreated *vs.* 0.62 pretreated). Finally, the mass balance showed that 38.2 kg glucose and 13.1 kg xylose were converted into 12.5 kg of methane per 100 kg of untreated ATB, representing 86% of the theoretical methane yield and evidencing the potential of this biorefinery scheme.

## Abstract 2

Methane conversion is enhanced by optimized bioderived ionic-liquid pretreated *Agave tequilana* bagasse with in-depth biomass characterization analysis.

## Graphical abstract

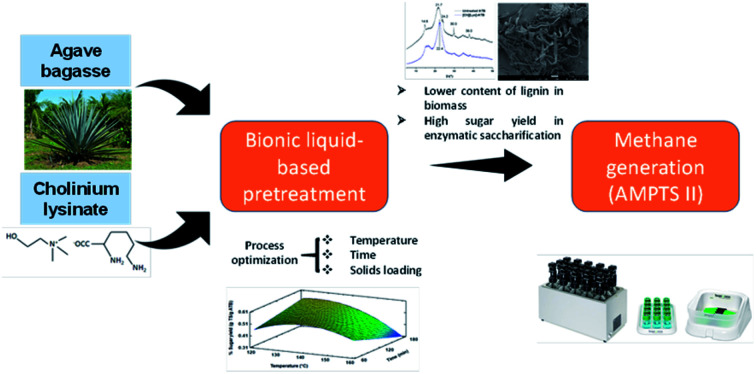

## Introduction

Increased industrialization and deforestation combined with the enhanced price of conventional fuels have led to depletion of feedstock for first generation biofuels (such as: sugars, starch, animal fats, and vegetable oils).^1^ On the other hand, lignocellulosic biomass (LCB) is the most available carbon resource generated on earth and is considered a safe alternative to petroleum-based fuels, equivalent to zero emission and a renewable energy resource for the production of biofuels and bioproducts.^2^

In recent years, *Agave* has emerged as a potential bioenergy feedstock in Mexico due to their advantageous features (*e.g.* low water requirements, high productivity up to 44 ton per ha per year, and drought resistance) with an estimated productivity of 11.4 × 10^5^ tons in 2018 for *Agave tequilana*, from which approximately 40% were converted to bagasse.^3–5^ Agave bagasse as any other LCB is constituted of a cross-linking matrix of lignin and hemicelluloses that embeds the cellulose fiber making it highly recalcitrant for biological attack.^6^ For this reason, a pretreatment step must be implemented to overcome the LCB recalcitrance and achieve an efficient biochemical conversion.

Up to now, different but limited single or combination of physical, chemical, physicochemical and biological pretreatment methods have been used in Agave bagasse. The efficiency and productivity of these studies can be consulted in a review elsewhere.^7^ Specifically, for gaseous biofuels (methane and hydrogen) generation from *Agave tequilana* bagasse (ATB), only alkaline hydrogen peroxide and dilute acid pretreatments have been implemented, obtaining 0.35 and 0.28 L CH_4_ per g COD (chemical oxygen demand), respectively.^8–10^

All the pretreatment methods notably alter the chemical and physical structure of lignocellulosic biomass with their specific mode of action, which enhances subsequent saccharification and fermentation steps.^11^ Ionic liquid (IL) pretreatment has been successfully implemented in ATB with promising results to achieve a suitable, efficient and cost-effective technology. ILs are salts with low meting points and favorable solvent properties such as non-flammability, low or negligible vapor pressure, chemical and thermal stability.^12^ The main effects of IL pretreatment on ATB are the partial removal of hemicellulose/lignin and crystalline cellulose without the generation of inhibitors or cellulose degradation, which entails an efficient saccharification and biofuel generation.^13^

To the best of our knowledge, all of the reports regarding IL pretreatment on ATB are imidazolium-based ILs, such as 1-ethyl-3-methylimidazolium acetate [Emim][OAc],^14–17^ 1-butyl-3-methylimidazolium chloride [Bmim][Cl]^18^ and 1-butyl-3-methylimidazolium acetate [Bmim][OAc].^19^ However, imidazolium-based ILs are not compatible and exhibit toxicity to enzymes and microbes, making necessary to implement an extensive water wash process to remove the residual IL after pretreatment. Recently, the synthesis of novel ILs from renewable biomaterials, such as the bioderived cholinium ILs have gained attention as new promising approaches due to their biocompatibility.^20,21^

Different reports have demonstrated the potential of cholinium lysinate ([Ch][Lys]) that derives from amino acids as a sustainable and less toxic IL for LCB pretreatment with comparable or higher efficiencies to non-renewable ILs in terms of delignification capability, enhancing the accessibility of polysaccharides to enzymes and retaining effectiveness during reuse.^22–25^

Due to the aforementioned, we aimed to evaluate for the first time the effect of [Ch][Lys] pretreatment on ATB and further methane production *via* anaerobic digestion. A central composite design (CCD) was employed to optimize the pretreatment conditions followed by in-depth biomass characterization and methane generation from pretreated solids and enzymatic hydrolysates. Briefly, pretreatment conditions (temperature, time and solids loading) were optimized for maximizing sugar production during the saccharification step. Then, recalcitrance parameters (*i.e.* lignin and crystallinity) were measured in the untreated and IL pretreated biomass to evaluate the effect of pretreatment on the plant cell wall using X-ray diffraction, Fourier transform infrared (FTIR) and confocal fluorescence/scanning electron microscopy. Finally, we provide a mass balance using the optimized pretreatment conditions based on 100 kg of untreated ATB using the glucan and xylan conversion and methane production by anaerobic digestion.

## Experimental

### Experimental design

A preliminary factorial experimental design was carried out in order to set an adequate solid loading for the further optimization experimental design. Six different IL pretreatment conditions were evaluated based on previous reports of different biomass feedstocks.^21,24,26^ Thus, the ATB was subjected to pretreatment with the renewable IL [Ch][Lys] using a fixed time of 120 min, two temperatures (120 °C and 140 °C) and three solids loading (10, 20 and 30%). The main response variable was the sugar yield [g total sugars (TS) per g ATB] obtained during the enzymatic saccharification of pretreated solids (Table 1).

**Table 1:** Experimental data from the preliminary runs of [Ch][Lys]-pretreatment

Run	Condition	% Solids recovery	Total sugars (g TS L^−1^)	% Sugar yield (g TS per g ATB)
A	140 °C, 10%	56.7	38.9 ± 0.7	44.9 ± 0.2
B	140 °C, 20%	67.9	38.8 ± 0.8	53.8 ± 0.2
C	140 °C, 30%	78.4	29.4 ± 0.1	48.3 ± 0.1
D	120 °C, 10%	62.2	32.8 ± 0.2	42.9 ± 0.2
E	120 °C, 20%	71.6	33.5 ± 0.6	51.0 ± 0.6
F	120 °C, 30%	77.0	27.0 ± 0.6	44.3 ± 0.4

Optimization of the temperature and reaction time of the IL pretreatment to maximize sugar yield during enzymatic hydrolysis was carried out by CCD as reported elsewhere.^17^ In this second design, the solids loading was set at 20% since it was the best condition found during the preliminary experimental design (Table 1). Therefore, the CCD consisted on 4 factorial, 3 central and 4 axial points (Table 2); the data was analyzed using the Statgraphics Centurion XV software including the response surface plotting.

**Table 2:** Experimental data from pretreated ATB using [Ch][Lys] at different pretreatment conditions from the CCD

Run	Condition	% Solids recovery	Total sugars (g TS L^−1^)	% Sugar yield (g TS per g ATB)	% Glucan conversion	% Xylan conversion
1	120 °C, 60 min	78.4	29.1 ± 0.0	47.0 ± 0.5	66.7 ± 0.8	68.2 ± 0.4
2	160 °C, 60 min	70.0	36.3 ± 0.1	52.3 ± 0.1	74.8 ± 0.1	73.7 ± 0.2
3	120 °C,180 min	71.0	35.2 ± 0.5	51.7 ± 0.1	73.9 ± 0.1	73.4 ± 0.1
4	160 °C, 180 min	113.4	15.0 ± 0.4	34.7 ± 0.4	50.0 ± 0.7	47.5 ± 1.6
CP1	140 °C, 120 min	67.9	38.8 ± 0.8	53.8 ± 0.2	79.9 ± 0.7	65.9 ± 3.6
CP2	140 °C, 120 min	69.4	37.2 ± 0.1	52.6 ± 0.2	75.3 ± 0.3	73.8 ± 0.7
CP3	140 °C, 120 min	68.9	36.3 ± 0.1	51.3 ± 0.1	74.0 ± 0.8	70.1 ± 1.8
1A	112 °C, 120 min	76.4	28.8 ± 0.5	46.2 ± 0.9	67.3 ± 1.4	60.9 ± 1.0
2A	168 °C, 120 min	151.3	6.8 ± 0.0	21.4 ± 0.0	33.2 ± 0.1	21.4 ± 0.0
3A	140 °C, 35 min	73.7	33.2 ± 0.3	50.0 ± 0.4	71.3 ± 0.6	71.4 ± 0.4
4A	140 °C, 205 min	71.7	36.2 ± 0.1	52.4 ± 0.1	76.8 ± 0.6	67.5 ± 1.3

### Materials and sample preparation

ATB was received from a tequila factory located in Jalisco, Mexico denominated Casa Herradura. In brief, after removing all leaves from the *Agave* in a traditional tequila process, the *Agave* stems are cooked during 24 h at 90–100 °C in a brick oven, milled and pressed to separate the liquid fraction (must) used for spirits production, while the solid fraction (bagasse) is used for the purpose of this study. A washing step was performed in ATB to remove any possible residual sugars, sun-dried for 3 days and particle reduced in a Pulvex mill (Pulvex Plastic, Mexico City, Mexico) equipped with a 20-mesh screen sieve and stored at 4 °C before use. The ionic liquid cholinium lysinate ([Ch][Lys)] was prepared and characterized as described in ESI Fig. S1.†

### Ionic liquid (IL)-based pretreatment

ATB samples were pretreated with [Ch][Lys] at the desired process conditions [solids loading (% w/w), temperature (°C) and reaction time (h)] accordingly to the corresponding design. Each pretreatment run was carried out in a convection oven where IL and ATB solids (in dry basis) were mixed to generate a slurry in a 250 mL glass reactor, then heated to the temperature and time selected.

After pretreatment, a washing stage was carried out to remove lignin and IL; the pretreated solids were recovered as previously described.^15^

### Enzymatic saccharification

Saccharification of raw and IL-pretreated ATB were performed using 4% solids loading in 50 mM citrate buffer at pH 4.8. Commercial enzymatic cocktail from Novozymes, CTec2 with an activity of 161 FPU per mL was employed. An enzymatic loading equivalent to 8 FPU per g solids was used for the runs of the experimental designs. The reaction conditions were set at 50 °C and 150 rpm during 72 h in a rotary incubator.

All assays were performed in duplicate and the sugar production was followed by sampling 1 mL at 0, 1, 2, 4, 8, 24, 48 and 72 h.

### Anaerobic digestion experiments

The inoculum employed (anaerobic granular sludge) was obtained from a full-scale up-flow anaerobic sludge blanket (UASB) reactor treating tequila vinasses. Batch assays were carried out using an inoculum concentration of 10 g L^−1^ of volatile solids (VS).

All reactions were performed in an automatic methane potential test system (AMPTS II, Bioprocess Control, Lund, Sweden) that consists of fifteen single 0.5 L reactors, each one with their individual mixing motor, CO_2_ elimination unit and CH_4_ flow measurement cell. During the experimental runs, the temperature, pressure and accumulated gas volume were recorded automatically by the AMPTS II system; at the end of the process a report was generated with normalized values of flow and accumulated gas.

These experiments were carried out at 37 °C, with an agitation of 60 rpm and a 360 mL working volume supplemented with the substrate and a mineral medium adjusted to pH 8 as previously reported by Arreola-Vargas *et al.*^9^ The substrate for the anaerobic digestion experiments were: enzymatic hydrolysates obtained from saccharification of pretreated ATB at a concentration of 5 g TS L^−1^ and the pretreated ATB solids at 5 g VS L^−1^ (both obtained at optimal [Ch][Lys]-pretreatment conditions); comparison with untreated ATB solids was evaluated. Experiments were carried out in triplicate.

### Analytical methods

The main cell wall components (cellulose, hemicellulose and lignin) of raw and pretreated ATB were determined using a semiautomatic fiber analyzer (ANKOM Technology, Macedon, NY, USA).^27^ TS in the enzymatic hydrolysates were measured as reported by DuBois *et al.*^28^ and monosaccharides were determined as previously reported.^17^

Volatile solids from ATB and granular sludge were determined according to standard methods.^10^ The chemical oxygen demand (COD) was obtained using the standard method of APHA 5220 using vials TNT 822 in a DRB200 digester and a DR2800 spectrophotometer.^10^ Attenuated total reflectance (ATR)-FTIR and X-ray powder diffraction (XRD) patterns of untreated and pretreated samples were obtained as previously reported.^17^ Crystallinity index (CrI) were determined according to Segal *et al.*:^29^
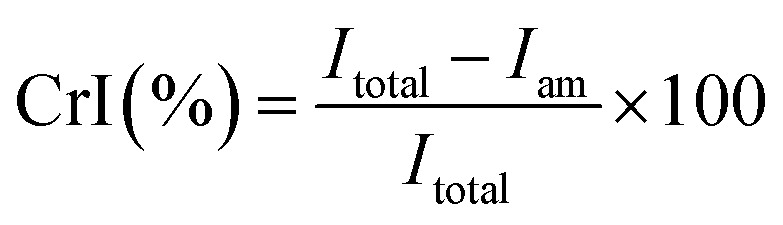


Finally, morphology of raw and IL-pretreated samples were analyzed by scanning electron and confocal fluorescence microscopy as reported by Avila-Lara *et al.*^30^

All the statistical analysis were carried out by using Statgraphics Centurion XV software.

## Results and discussion

### Effect of solids loading on sugar pretreatment

In order to build an adequate and precise design to carry out the pretreatment optimization, preliminary experimental runs at different solids loadings, two temperatures and at a fixed time of 120 min were evaluated in terms of sugar yield.

Table 1 shows that in both temperatures, an increase in the solids recovery was attained accordingly to the solids loading during pretreatment from 10 to 30%. A similar effect was reported for the pretreatment of corn stover at 140 °C with [Ch][Lys], in which the solids loading at 50% (w/w) generated a solids recovery of 65% while at 15% a recovery of 46% was obtained.^23^

**Fig. 1:**
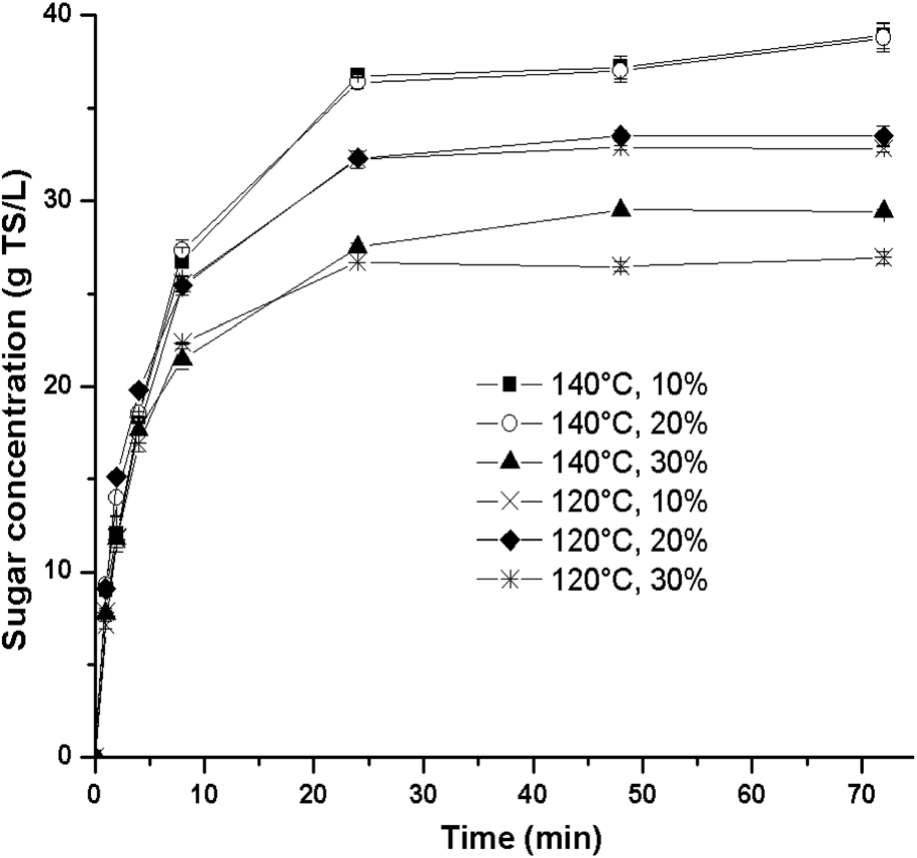
Kinetic profiles of sugar concentration obtained from the first experimental design at different conditions of temperature and solids loading.

Regarding sugar release during saccharification, Fig. 1 shows that the two highest TS concentrations were accomplished at 140 °C, where the solids loading factor did not have a significant effect. However, at a solids loading of 30%, the concentration of sugars in the hydrolysate decreased for both temperatures to 27.0 g TS L^−1^ (120 °C) and 29.4 g TS L^−1^ (140 °C). Thus, the best sugar yield was obtained in experimental run B at a solids loading of 20%, achieving 53.8 g TS g^−1^ ATB (Table 1). Interestingly, this sugar yield is similar to the optimum one found in a recent report with [Emim][OAc] (52.1 g TS g^−1^ ATB at 120 °C, 120 min and 30% solids loading);^17^ however, the pretreatment conditions are different. In this sense, the experimental run F shows that at the same optimal [Emim][OAc]-pretreatment conditions, the renewable IL [Ch][Lys] produced a lower sugar yield of 44.3%. Therefore, it is clear that the chemical nature of each specific IL generates different responses.

### Optimization of the pretreatment conditions

From the proposed CCD design, 11 experimental conditions were evaluated including 3 central points for measuring the experimental error (Table 2). The solids loading was fixed at 20%.

According to the nature of the CCD design, the runs of the 2^k^ factorial design along with the central points were firstly executed and once lack of fit to a linear model was found (*p* = 0.0227), the axial points were added to fit the data to a second order model and find an optimal response.^17^

Interestingly, a special phenomenon occurred under conditions 4 and 2A where solid recoveries surpassed 100%. This suggests that solid compounds were formed from [Ch][Lys] due to the high pretreatment temperatures (160 °C and 168 °C). The [Ch][Lys] decomposition temperature is 168 °C but some studies suggest that ILs decomposition occur at temperatures close to the decomposition one.^31,32^ According to this information, a hypothesis could be formulated in which the combination of a long reaction time (180 min) with the temperature of 160 °C, generated a lower thermostability of [Ch][Lys], causing its degradation in solid compounds that impacted the recovery of solids. Apparently, this did not happen with a lower reaction time of 60 min, since their recovery of solids (70%) and concentration of sugars during saccharification (36.3 g TS L^−1^) are similar to the best condition of the design (120 °C and 180 min) with 71% and 35.2 g TS L^−1^, respectively.

**Fig. 2:**
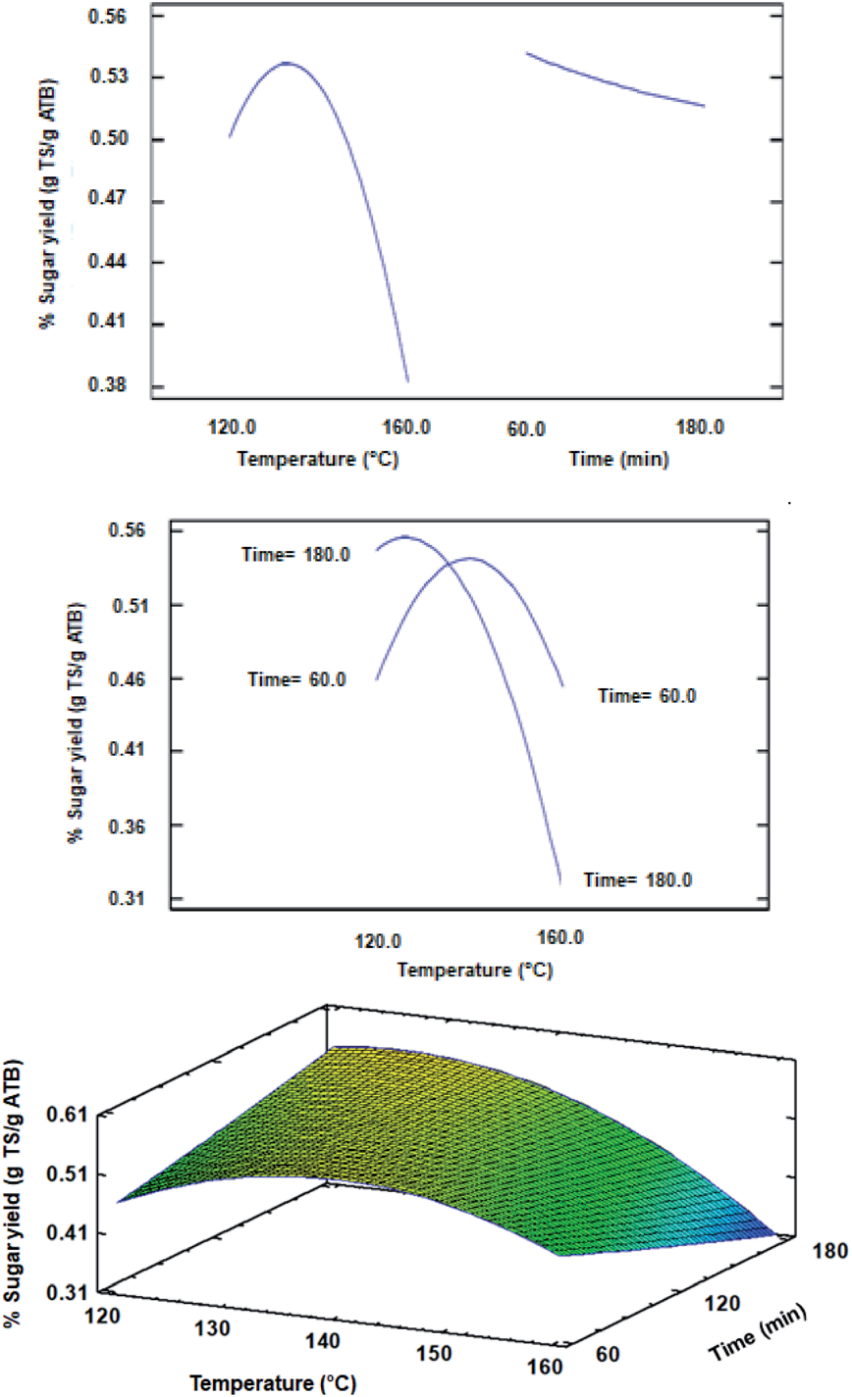
Main effect (top), interaction (middle) and response surface (bottom) plots obtained for CCD evaluating the temperature and time effects on sugar yield.

The ANOVA of the CCD indicated that the significant factors of the design were the temperature and the quadratic factor of temperature, with a statistical confidence of 95% (data not shown). The time did not significantly influence the TS yield for the intervals that were evaluated in the design, which can be confirmed in Fig. 2. However, in the response surface plot from Fig. 2, it can also be clearly observed that at low temperatures (such as 120 °C), the TS yield is being favored as the reaction time increases. In this sense, Hou *et al.*, showed that for rice straw [Ch][Lys]-pretreatment at 90 °C, when the pretreatment time was greater than 5 h, its effect on enzymatic saccharification decreased although the extraction of lignin was improved.^22^ A similar effect could have happened during the conditions evaluated in the current experimental design, taking into consideration that a similar pretreatment severity with relatively high temperatures (greater than 90 °C) and short residence times (less than 180 min) could have generated a similar effect to the one reported by Hou *et al.*^22^

As regards to the optimal conditions for the pretreatment, the most suitable regression model obtained by eliminating non-significant terms with an *R*-square of 0.9780 is shown in the following equation:*Y* = 0.0101991*X* − 0.0000482869*X*^2^where *Y* represents the yield of total sugars (g TS per g ATB) and *X* the temperature. The optimal pretreatment conditions determined by the model are a temperature of 124 °C and a reaction time of 205 min. Similarly to [Emim][OAc]-pretreatment, the mild conditions in ATB to maximize sugar production contrast with the typically harsher conditions (140–160 °C) applied in other feedstocks such as corn stover or switchgrass that ultimately must be taken into consideration in the total production costs in a biorefinery scheme.^23,33^

### Effects of pretreatment on the *Agave tequilana* bagasse (ATB) cell wall composition and structure

The main constituents of the plant cell wall of ATB were glucan (54.4%), xylan (15.7%) and lignin (12.1%). While ATB was consistent with previous reports in terms of xylan and lignin, the glucan content was relatively high, which can be attributed to the environmental conditions and post-harvest procedures including the cooking conditions during tequila production.^34^

According to the optimal conditions obtained in the preceding subsection, [Ch][Lys]-pretreatment of ATB was carried out. The solids recovery from the pretreated biomass was 75.0% with a composition of 68.9% glucan, 18.0% xylan and 8.8% lignin. Thus, the main observed changes in the cell wall composition of pretreated ATB were an increase in glucan/xylan content and lignin removal. Taking into consideration the solid recovery, the delignification obtained was 45.4%, demonstrating the high capacity for the removal of lignin. In addition, the removal of lignin by [Ch][Lys] is due to the anion lysinate, whose delignification capacity is favored when a temperature higher than 90 °C is used.^35^

**Fig. 3:**
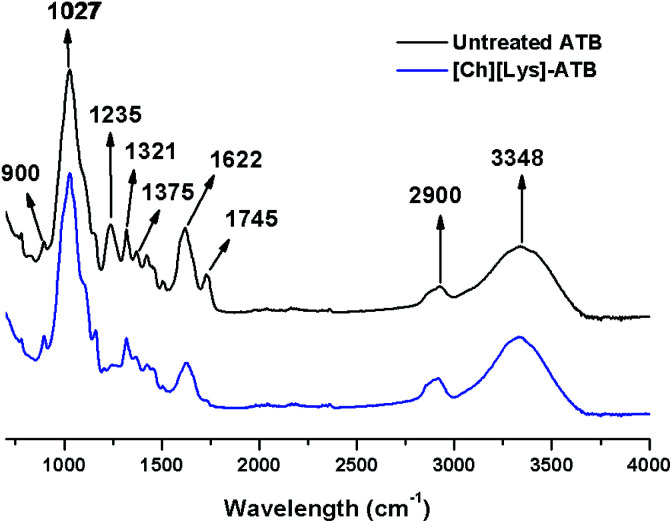
Chemical changes in ATB solids (untreated and IL-pretreated) determined by FTIR-ATR.

The chemical changes after pretreatment were followed by seven FTIR bands of carbohydrate and lignin plus two bands related to calcium oxalate. According to Fig. 3 and ESI Table S1,† a minimal change in the band of amorphous cellulose (900 cm^−1^) occurred, possibly due to a slight modification on cellulose crystallinity. Simultaneously, a strong effect on the C–O stretching in lignin and hemicellulose (1235 cm^−1^) due to the relatively high lignin removal occurred after pretreatment.^36^ Furthermore, an elevated increment to 68.1% in the 1745 cm^−1^ band [carbonyl (C=O) stretching] indicate cleavage of lignin and side chains were obtained. In addition, a reduction in the intensities of the bands 2900 and 3348 cm^−1^ occurred in the pretreated ATB when compared to the untreated solids, which could indicate a contraction of the C–H and O–H linkages that contrast with other pretreatments such as lime or dilute acid.^37^

A significant reduction in the band positions attributed to calcium oxalate at 1321 cm^−1^ and more noticeable at 1622 cm^−1^ was obtained in the [Ch][Lys]-pretreated ATB, which is higher than the changes obtained with [Emim][OAc]-pretreated ATB.^34^ In addition, the cellulose crystallinity index (CrI) is an important parameter to evaluate the response on enzymatic saccharification.

The powder XRD patterns of untreated and [Ch][Lys]-pretreated ATB are shown in ESI Fig. S2.† The CrI values of untreated and IL-pretreated ATB were 0.51 and 0.62, respectively. It is noticeable, that unlike imidazolium-based IL pretreatment in ATB, where a reduction of cellulose crystallinity occurred, an increase in the CrI values were attained.^15,17^ This effect of increasing the CrI after the [Ch][Lys] pretreatment of LCB has been previously reported and was attributed to the removal of the amorphous cell wall components such as lignin and hemicellulose.^21,22^ Finally, scanning electron and confocal fluorescence microscopy high-resolution images from untreated and [Ch][Lys]-pretreated ATB are shown in ESI Fig. S3 and S4,† respectively. It can be observed in ESI Fig. S3† that after pretreatment the samples have swollen structures, with broken fibers including amorphous and irregular parts. This causes an increase in the surface area that has been reported as positive in the pretreatment of rice straw using [Ch][Lys] mixed with water.^38^ The phenomenon of LCB structure swelling could be due to the breakdown of the lignin-carbohydrate bonds, which occurs after lignin removal during the dissolution and regeneration process of the cellulose.^34^ Besides, calcium oxalate monohydrate crystals were found in untreated and [Ch][Lys]-pretreated (ESI Fig. S3†).

The oxalate crystals found in the BAT have a prismatic and regular shape while those of the [Ch][Lys]-pretreated ATB are prisms with irregular structure and rounded tips. The formation of spherical crystals can be an effect of the IL pretreatment and this tendency increases the greater the severity of the pretreatment.^39^ Additionally, the elemental composition (using EDS) that was obtained for the oxalate crystals in the ATB and [Ch][Lys]-pretreated ATB are similar to each other and approximates to the theoretical value obtained by the compound formula (ESI Table S2†).

In the ESI Fig. S4† is presented the micrographs taken by confocal microscopy of the untreated and [Ch][Lys]-pretreated ATB in which distinctive fluorescence intensities are observed for lignin (blue), cellulose (green) and hemicellulose (red). Interestingly, minor delignification was observed in the pretreated solids with [Ch][Lys].

This could be attributed to the fact that pretreatment with [Ch][Lys] generated a swelling effect and an increase in the surface area of lignin, which caused this component to visually appear a greater presence in the lignocellulosic matrix when in fact a considerable part of the lignin was removed by the action of the pretreatment.^17,34^

### Methane production from enzymatic hydrolysates and pretreated solids obtained at optimal pretreatment conditions

Anaerobic digestion is an attractive process for the production of gaseous biofuels because it is carried out by microbial communities, which present a high diversity of metabolic pathways and in turn provide robustness to the process.^40^ However, the metabolic capacity of these communities is insufficient to efficiently incorporate the nutrients that make up the ATB without pretreatment.

Due to the fact that IL pretreatment modify the lignocellulosic structure making it less recalcitrant and more accessible to the microbial degradation, during the anaerobic digestion assays not only the enzymatic hydrolysates but also the solids from the [Ch][Lys]-pretreated ATB were evaluated as substrates.

**Fig. 4:**
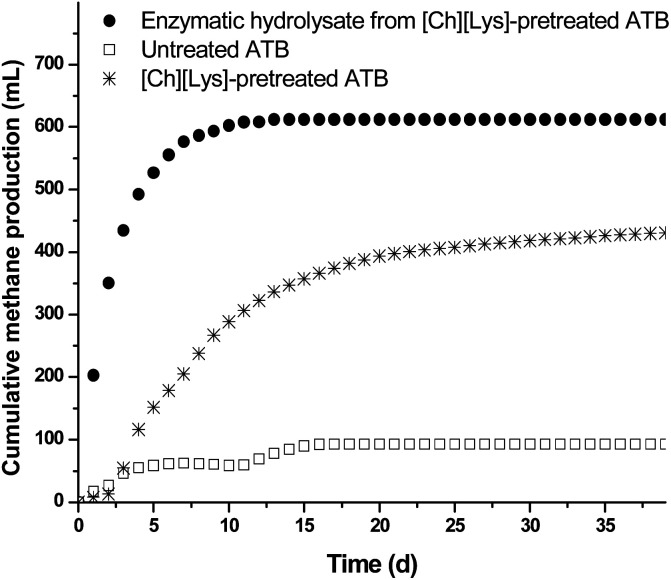
Methane production kinetics from enzymatic hydrolysate and solids of ATB (untreated and pretreated with [Ch][Lys]).

Fig. 4 shows the kinetic profiles for methane production from enzymatic hydrolysates and pretreated ATB solids (both obtained at optimal [Ch][Lys]-pretreatment conditions); the methane production from the untreated ATB is also shown for comparison purposes. It is clearly noticeable that the hydrolysate achieved the highest accumulation of methane up to 613 mL with a yield of 0.30 L CH_4_ per g COD (86% of the theoretical maximum yield of 0.35 L CH_4_ per COD), whereas the lowest methane was produced by the untreated ATB reaching only 80 mL and a yield of 0.04 L CH_4_ per COD. The methane yield obtained with this type of hydrolysates is similar to other reports using specialized reactors or similar hydrolysates.^41,42^

Interestingly, the [Ch][Lys]-pretreated solids without being saccharified reached 433 mL of methane with a yield of 0.24 L CH_4_ per VSS, representing a 5.4 fold compared to the untreated ATB and a 70% of the methane produced by the hydrolysate; making clear that the availability of the nutrients contained in the ATB increases considerably through pretreatment with ILs.

It is important to mention that the values reported for methane production were calculated by subtracting the values obtained with the endogenous and enzyme controls in order to eliminate the contribution of COD from the inoculum and the enzyme to the process as previously reported.^9^ Even though the [Ch][Lys]-pretreated solids generated a lower amount of methane compared to the hydrolysate and also a lower methane production rate was observed (0.09 *vs.* 0.43 L CH_4_ per L per d), the scarce reports on anaerobic digestion using IL pretreated LCB solids (Table 3) show that the reported yield in this work is the highest reported so far with a value of 0.24 L CH_4_ per g VSS, confirming the potential of both the type of LCB and IL pretreatment.^43–45^

**Table 3:** Comparison to reported works on anaerobic digestion of different IL pretreated biomass

Biomass	IL pretreatment	Yield (L CH_4_ per g VSS)	Reference
ATB	[Ch][Lys]	0.24 ± 0.12	This study
Water hyacinth	[Bmim][Cl]/DMSO^a^	0.16	43
Water hyacinth	[Bmim][Cl]	0.20	44
Grass	[Bmim][OAc]	0.22	45

a DMSO: dimethyl sulfoxide.

Finally, regarding the hydrolysis step during the anaerobic digestion of ATB solids, the degradation of lignocellulose in these systems has been reported to be carried out by microorganisms such as the genus *Acetivibrio* and *Clostridium*,^46^ so it is possible that microorganisms of this type have participated in the hydrolysis of [Ch][Lys]-pretreated ATB, since they have been reported in previous works using the same inoculum.^41^

### Mass balance of optimized pretreated-*Agave tequilana* bagasse for methane production

Based on the optimum pretreatment conditions, hydrolysates from [Ch][Lys]-pretreated ATB were obtained and characterized, presenting high COD (35.4 g L^−1^) and concentration of total sugars (32.5 g L^−1^) constituted by 16.1 g glucose per L, 7.4 g xylose per L among others sugars. Noticeable it is the absence of inhibitory compounds for microorganisms such as furfural, hydroxymethylfurfural (HMF), and acetic acid.^47^ It is important to point out that a considerable part of the sugars obtained are pentoses, which represents 22.7% of the TS in the hydrolysate. The utilization of xylose can be carried out by microorganisms that participate in anaerobic digestion, for which hydrolysates from [Ch][Lys]-pretreated ATB are a potential substrate for this process.^48^

Total sugars production from saccharification of [Ch][Lys]-pretreated ATB reached 32.5 g TS L^−1^ in 72 h with a 6.3-fold increase when compared to the untreated biomass (5.2 g TS L^−1^) and an accelerated kinetics between 10 and 48 h (data not shown).

This high sugar concentration along with a high yield (51.4 g TS per g ATB) occurred by different reasons. First, a high solids recovery during [Ch][Lys] pretreatment; and second, a high lignin removal (45.4%) including the modification of the carbohydrate-lignin linkages as shown by FTIR. However, a high delignification does not always guarantee an efficient enzymatic saccharification. For instance, during the AHP pretreatment of ATB, a higher delignification was achieved (97%) compared to [Ch][Lys] pretreatment (45.4%); however, a 3-times lower TS concentration was obtained with AHP pretreated ATB.^8^

**Fig. 5:**
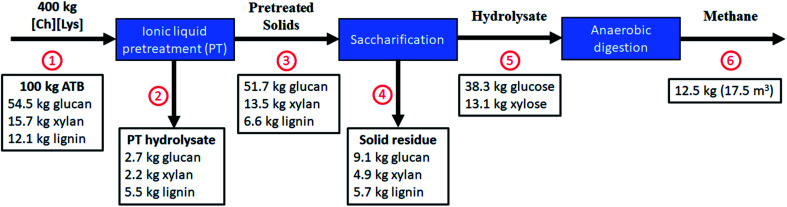
Mass balance per 100 kg of untreated ATB in dry weight using optimized pretreatment conditions with high solids loading for sugars and methane production. (1) ATB input; (2) liquid waste from pretreatment; (3) pretreated ATB; (4) non-saccharified solids; (5) enzymatic hydrolysate; (6) methane production.

In order to establish a mass balance of the bioprocess transformation of ATB into methane to gain a better understanding of [Ch][Lys] pretreatment technology, the compositional analysis, sugar yield, and methane production were normalized into a 100 kg ATB basis in dry weight (Fig. 5).

Using the optimized pretreatment conditions (124 °C, 205 min, and 20% solids loading), a solid recovery of 75.0 kg was obtained (including: 51.7 kg of glucan and 13.5 kg of xylan) after a water wash step where mostly lignin was solubilized into the liquid stream. These washes have the purpose of removing [Ch][Lys] from the biomass, so it does not interfere during the enzymatic saccharification and it can be recovered for reuse. It has been reported that [Ch][Lys] can be reused up to 5 times, achieving glucose yields higher than 80%, making this stage of the process critical for its economic feasibility.^22^

Moreover, taking into consideration the high recyclability of cholinium based ILs, these chemicals potentially meet environmental requirements for the development of cost-effective IL pretreatment technology in order to reduce up to 70–85% of greenhouse emissions.^25^

The pretreated solids showed a higher glucan content (51.7 kg) with a considerable decrease on lignin from 12.1 to 6.6 kg when compared to the untreated biomass. After the saccharification step, the optimized pretreated solids reached up to ∼51.4 kg of sugars per 100 kg untreated ATB. This sugar yield is higher than our previous reports where [Emim][OAc]-pretreated ATB achieved up to 29.2–44.6 kg sugars per 100 kg untreated ATB^4,34^ or from AFEX-pretreated ATB with 36.2 kg sugars per 100 kg untreated ATB.^49^

Additionally, this is the first report that carried out a process mass balance for methane production using ATB. After being anaerobically digested, the 51.4 kg TS of the enzymatic hydrolysate from [Ch][Lys] pretreated ATB were converted to 17.5 m^3^ of methane, equivalent to 12.5 kg CH_4_ per 100 kg untreated ATB. Such methane generation represents an energy recovery of 6.27 kJ g^−1^ ATB, which doubles the energy production reported by Arreola-Vargas *et al.* (2016) by using acid and enzymatic hydrolysates in one stage anaerobic digestion and it is similar to the energy recoveries reported in two-stage anaerobic digestion processes.^9^

## Conclusions

Optimized [Ch][Lys]-pretreated ATB (124 °C, 205 min, 20% solids) was able to achieved 51.4 kg of sugars and 12.5 kg of CH_4_ per 100 kg of untreated biomass due to its relatively high delignification (45.4%) and weaken chemical bonds. The highest methane generation was obtained using the hydrolysate from IL-pretreated ATB with a yield of 0.30 L CH_4_ per g COD, which is 7.5 times higher when compared to that obtained with the untreated ATB. The pretreated solids were also able to be highly anaerobically digested obtaining a yield of 0.24 L CH_4_ per g VSS, representing a promising approach within a biorefinery scheme since no saccharification is needed. Overall, this study presents an attractive approach to pretreat a residue of great concern in Mexico by applying a potential, economical and environmentally friendly pretreatment that improves the generation of methane.

## Conflicts of interest

There are no conflicts to declare.

## Supplementary Material

RA-010-D0RA01849J-s001
