# Supplementary material for: Bioderived ionic liquid-based pretreatment enhances methane production from Agave tequilana bagasse
Source: RSC Adv. 2020 Apr 7;10(24):14025–32. doi: 10.1039/d0ra01849j (PMC9051612; doi:10.1039/d0ra01849j)
Supplement: RA-010-D0RA01849J-s001 [file RA-010-D0RA01849J-s001.pdf]

Electronic Supplementary Material (ESI) for RSC Advances.
This journal is © The Royal Society of Chemistry 2020

ELECTRONIC SUPPORTING INFORMATION (ESI)

BIONIC LIQUID-BASED PRETREATMENT ENHANCES METHANE PRODUCTION FROM *AGAVE TEQUILANA* BAGASSE

Jose A. Pérez-Pimienta[a], José P. A. Icaza-Herrera[b], Hugo O. Méndez-Acosta[b], Víctor González-Álvarez[b], Jorge A. Méndoza-Pérez[c], Jorge Arreola-Vargas[d,*]

[a] Department of Chemical Engineering, Universidad Autónoma de Nayarit, Tepic, Mexico

[b] Departamento de Ingeniería Química, CUCEI-Universidad de Guadalajara, Guadalajara, Jalisco, México.

[c] Department of Engineering in Environmental Systems, Instituto Politécnico Nacional, Mexico City, Mexico

[d] División de Procesos Industriales, Universidad Tecnológica de Jalisco, Guadalajara, Jalisco, México.

*Corresponding author.

E-mail address: jorgearreolav85@gmail.com

ELECTRONIC SUPPORTING INFORMATION (ESI)

Table S1. Relative changes in ATB after IL pretreatment.

**Band**	**Assignment**	**% Relative change**
900	Anti-symmetric out of plane ring stretch of amorphous cellulose	-2.1
1027	C-O stretching in cellulose & hemicellulose	3.2
1235	C-O stretching in lignin & hemicellulose	48.1
1321	C=O stretching of calcium oxalate	13.8
1375	C-H deformation in cellulose & hemicellulose	13.9
1622	C-O stretching of calcium oxalate	42.9
1745	Carbonyl (C=O) stretching	68.1
2900	C–H stretching (related to rupture of methyl/methylene group of cellulose)	-18.5
3348	O–H stretching (indicates rupture of cellulose hydrogen bonds)	-6.8

%Relative change = 100 * [(intensity of untreated solids - intensity of pretreated solids)/intensity of untreated solids]; where positive numbers indicate reduction

Table S2. Elemental content from calcium oxalate crystals in untreated and [Ch][Lys] pretreated ATB.

Sample	Element	% Mass fraction	% Atomic mass fraction
**Untreated ATB**	C	10.8	18.0
	O	50.3	62.7
	Ca	38.9	19.3
**[Ch][Lys]-ATB**	C	13.5	23.0
	O	42.9	54.8
	Ca	43.6	22.2

ELECTRONIC SUPPORTING INFORMATION (ESI)

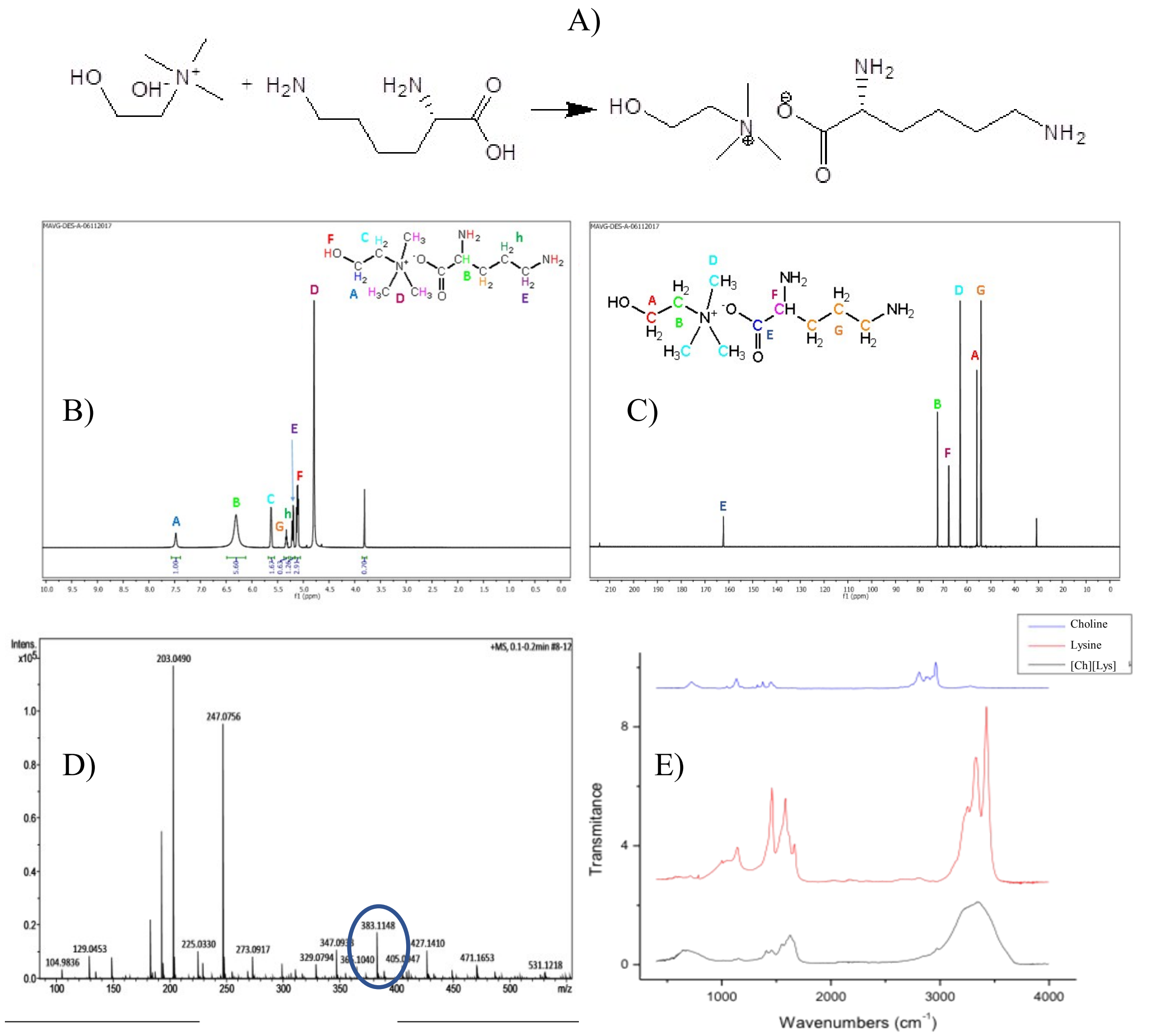

Fig. S1. A) Overall reaction to synthetize [Ch][Lys]*, B) ^{1}H-NMR spectra of [Ch][Lys] in D_2O, C) ^{13}C-NMR spectra of [Ch][Lys] in D_2O, D) Mass spectra of [Ch][Lys], and E) FTIR spectra of [Ch][Lys] and components.

*Synthesis was carried out according to Sun et al., *Green Chem.*, 2014, **16**, 2546–2557.

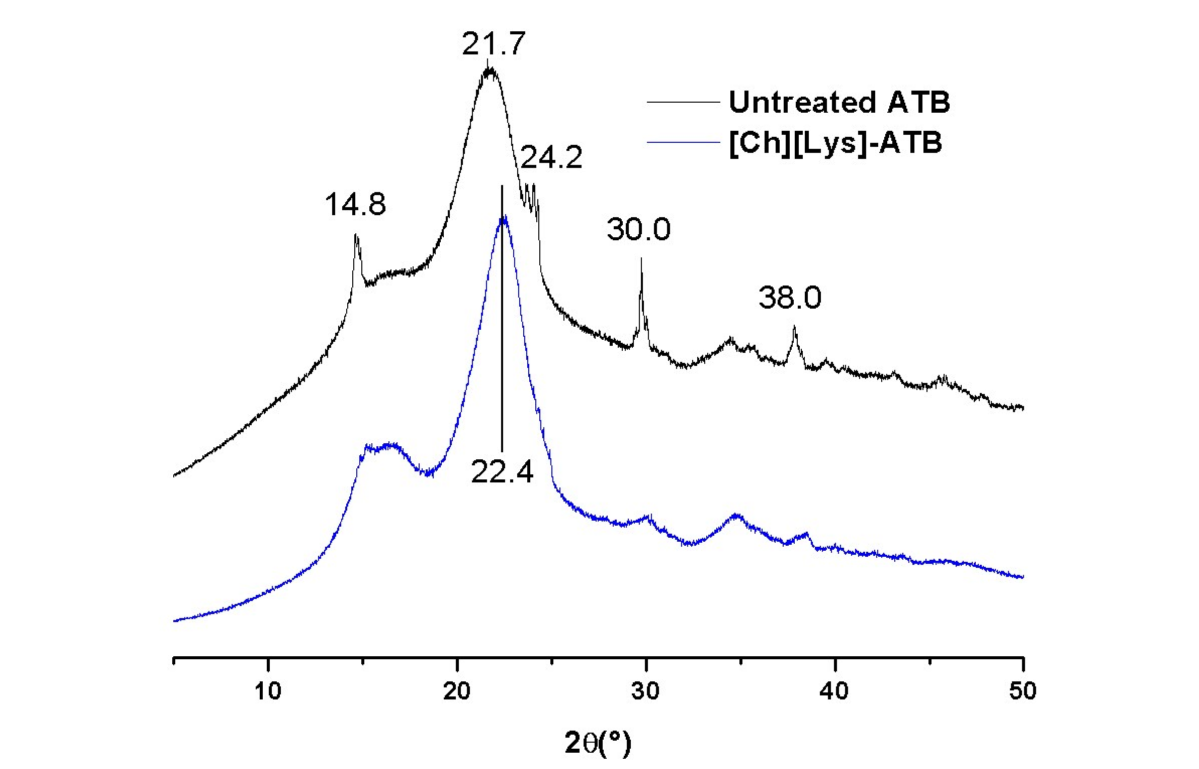

Fig. S2. XRD spectra of untreated and IL-pretreated ATB.

2

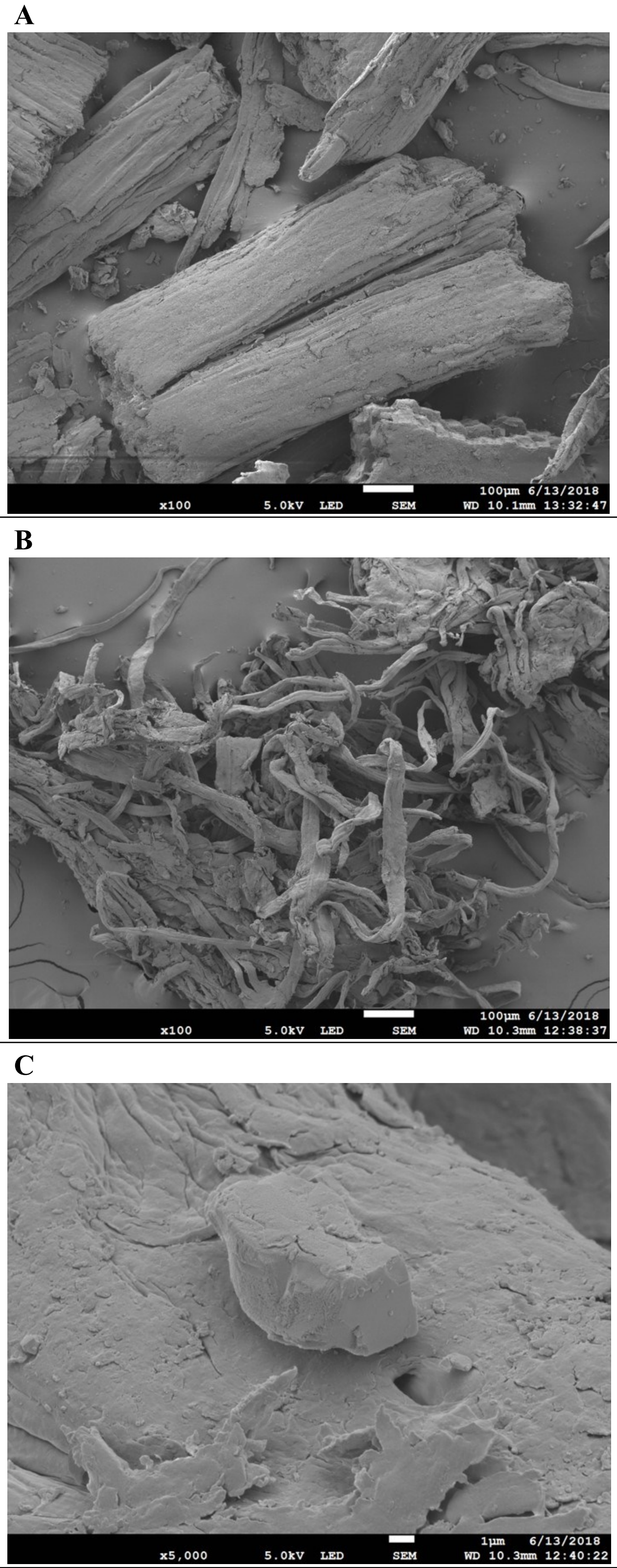

Fig. S3. Scanning electron microscopy images of untreated (A), [Ch][Lys]-ATB (B) and calcium oxalate crystals in pretreated ATB (C).

ELECTRONIC SUPPORTING INFORMATION (ESI)

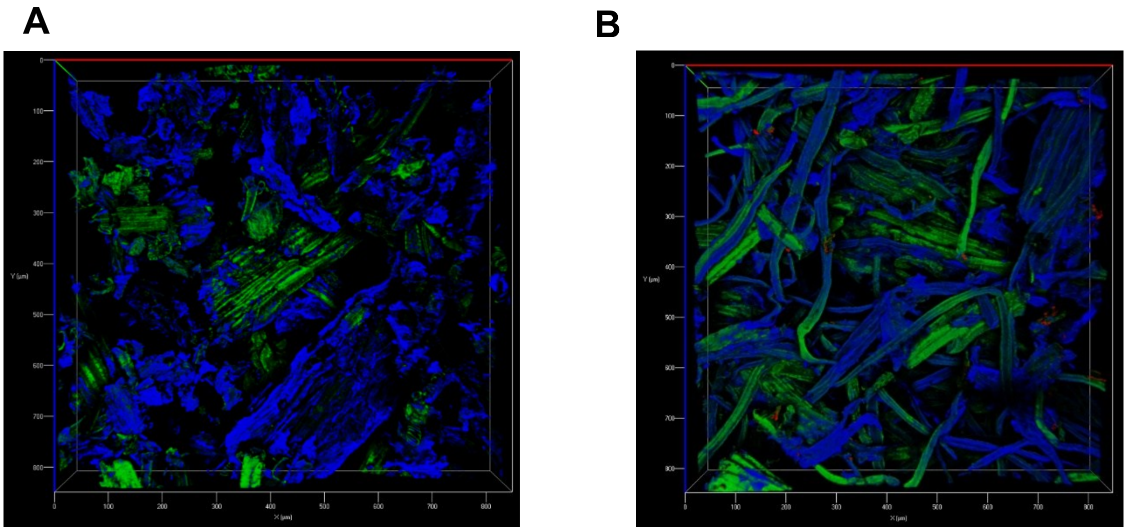

Fig. S4. Confocal fluorescence microscopy images of untreated (A) and [Ch][Lys] pretreated ATB.
